# Insights into the Transcriptomics of Crop Wild Relatives to Unravel the Salinity Stress Adaptive Mechanisms

**DOI:** 10.3390/ijms24129813

**Published:** 2023-06-06

**Authors:** Mughair Abdul Aziz, Khaled Masmoudi

**Affiliations:** Integrative Agriculture Department, College of Agriculture and Veterinary Medicine, United Arab Emirates University, Al Ain 15551, United Arab Emirates; 201350444@uaeu.ac.ae

**Keywords:** salinity, TFs, CWRs, transcriptomics, stress tolerance, genetic diversity

## Abstract

The narrow genomic diversity of modern cultivars is a major bottleneck for enhancing the crop’s salinity stress tolerance. The close relatives of modern cultivated plants, crop wild relatives (CWRs), can be a promising and sustainable resource to broaden the diversity of crops. Advances in transcriptomic technologies have revealed the untapped genetic diversity of CWRs that represents a practical gene pool for improving the plant’s adaptability to salt stress. Thus, the present study emphasizes the transcriptomics of CWRs for salinity stress tolerance. In this review, the impacts of salt stress on the plant’s physiological processes and development are overviewed, and the transcription factors (TFs) regulation of salinity stress tolerance is investigated. In addition to the molecular regulation, a brief discussion on the phytomorphological adaptation of plants under saline environments is provided. The study further highlights the availability and use of transcriptomic resources of CWR and their contribution to pangenome construction. Moreover, the utilization of CWRs’ genetic resources in the molecular breeding of crops for salinity stress tolerance is explored. Several studies have shown that cytoplasmic components such as calcium and kinases, and ion transporter genes such as Salt Overly Sensitive 1 (*SOS1*) and High-affinity Potassium Transporters (*HKTs*) are involved in the signaling of salt stress, and in mediating the distribution of excess Na^+^ ions within the plant cells. Recent comparative analyses of transcriptomic profiling through RNA sequencing (RNA-Seq) between the crops and their wild relatives have unraveled several TFs, stress-responsive genes, and regulatory proteins for generating salinity stress tolerance. This review specifies that the use of CWRs transcriptomics in combination with modern breeding experimental approaches such as genomic editing, de novo domestication, and speed breeding can accelerate the CWRs utilization in the breeding programs for enhancing the crop’s adaptability to saline conditions. The transcriptomic approaches optimize the crop genomes with the accumulation of favorable alleles that will be indispensable for designing salt-resilient crops.

## 1. Introduction

The agri-food systems are pressurized by the anthropogenic activities of poor land management and climatic changes in the form of salinity. These activities have resulted in the salinization of 20% of the irrigated lands, which incurs approximately USD 30 billion from the annual agricultural losses [1]. Such salt stress issues lead to the persistence of food scarcity around the world; however, its effects are detrimental specifically to the South Asian, Middle Eastern, and sub-Saharan African regions where agricultural production is limited the most [2]. This shows that salinity stress is a major obstacle to current and future global food production. Hence, addressing the issue of salinity will provide a compelling contribution to the economy of producers and to attaining the food demands needed to sustain the 10 billion people by 2050. This clearly makes it important to identify ways to alleviate the effects of salt stress by using saline soils and water to expand agricultural yield.

In order to utilize the salt-affected lands, it is essential to breed or engineer novel crop varieties that are tolerant to salinity, which can contribute to the sustainable intensification of agronomic systems. The degree of salt tolerance differs among plant species based on their genetic variations. Accordingly, all the available genetic diversity needs to be exploited and the current gene pools of the cultivated plants need to be expanded. One of the possible ways of achieving this solution is to explore the sources of wild desirable genes, such as from the crop wild relatives (CWRs) [3]. These crops are the ancestors or progenitors of domesticated plants that have higher genetic and phenotypic variability [4]. CWRs are present as wild species in their natural habitats or near their centers of origin [5]. Unlike the domesticated species, the wild relatives were not subjected to human selection pressure [6]. Instead, they developed and adapted in abundance throughout their evolutionary history and kept evolving in harsh climatic conditions including salinity. CWRs consist of a plethora of genes that are involved in their enhanced tolerance to salt stress, which represents a rich pool of alleles that are absent in the current cultivars with crucial agronomic value [6]. These resources when utilized are likely to broaden the genetic base of cultivated crops by introducing economically important genes, critical for meeting the challenges of food crisis and salinity stress.

Salt stress is a complex phenomenon under which CWRs have developed numerous adaptive responses, morphologically, physiologically, and biochemically. These responses are controlled at the molecular level by a large number of genes that signal, synthesize phytohormones, transport ions, and cause morphological changes [7]. To decipher these molecular mechanisms, transcriptomics is a powerful method to be employed [8]. Several global transcriptome profiling has been performed in salinity stress-adapted genotypes, but mainly in model plants [9]. Thus far, molecular mechanisms of CWRs salinity stress tolerance using transcriptomics have been rarely studied. As close relatives to various modern cultivars, a global transcriptomic investigation of salinity stress adaptation in CWRs can be very helpful. It will reveal the underlying molecular mechanisms of CWRs salt stress tolerance with the larger goal of incorporating them in the modern molecular breeding of crops.

In connection with this, the present study will indicate the importance of CWRs as a critical resource for the development of salinity-stress-resilient crops. This will ascertain their use for future food security, which has not been clearly recognized despite their capability as gene donors for crop enhancement. The combination of CWR breeding with the latest biotechnological advancements such as transcriptomics can be widely utilized for successfully generating salt-stress-tolerant plants. This is because coupling CWRs transcriptomics with in silico analysis can lead to an unbiased transcriptomic profile development for the identification and activation of certain physiological mechanisms, set of genes, and specified genetic pathways involved in plant salt stress tolerance.

Thus, to reveal the potential of CWRs’ salinity stress adaptation, a comprehensive understanding of their tolerance mechanism using transcriptomic approaches is required at present. In this aspect, the present review initiates by revisiting the impact of salt stress on plants. It recapitulates the salt stress responses at the molecular and phytomorphological levels for understanding the crop’s tolerance mechanism towards salinity stress. The present review highlights the importance of CWRs due to their wider genetic pool and harboring of TFs associated with the salt-stress-responsive genes. The transcriptome profiling and discerning of stress-tolerant genes in CWRs are further elaborated to improve their genetic resolution and use in modern breeding. The study suggests that transcriptomic approaches can offer unique insight into CWRs’ salt stress tolerance and drive the molecular breeding of crops for salinity stress tolerance.

## 2. Salinity Stress Impacts the Plant’s Growth and Physiological Processes

**Figure 1:**
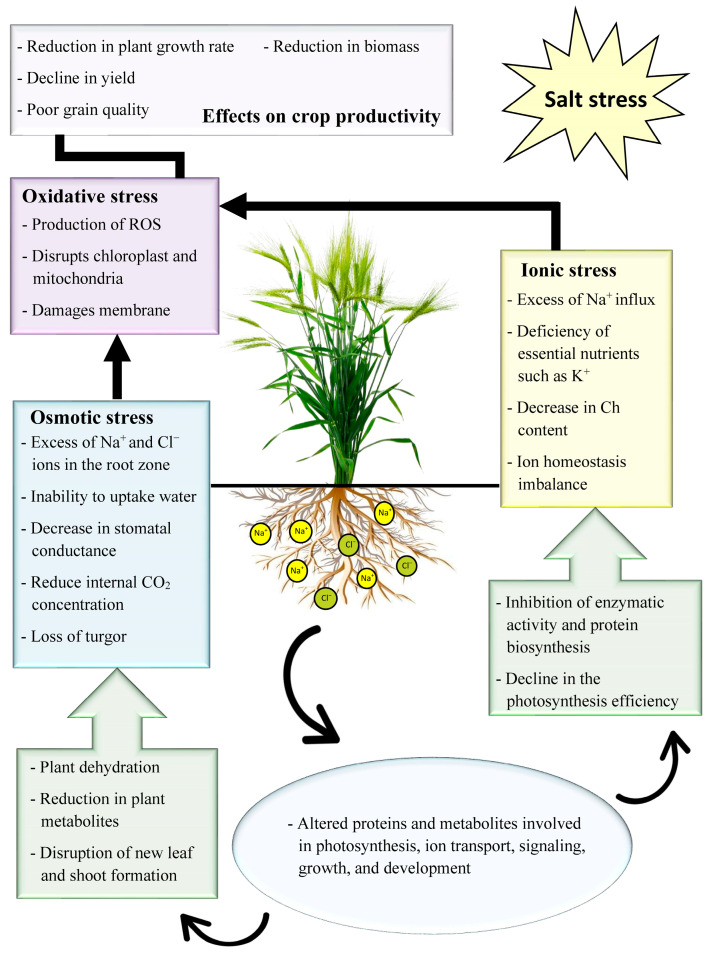
Overview of the effects of salinity stress on plants. The primary effects of the excess of salt ions in the soil or irrigation water cause ionic and osmotic imbalance due to restricted water and nutrient uptake due to altered proteins and metabolites. The build-up of salt ions within the plants generates ROS causing oxidative stress and disrupting the plant membrane and various organelles leading to declined growth and productivity.

Salt stress is a complex abiotic stress that substantially affects the plant’s productivity [10]. This stress occurs largely due to the high concentration of soluble salt ions within the soil solution that generates different stresses within the plants [10]. It lowers the soil water potential and restricts the plants from taking up water and nutrients. The entry of high levels of Na^+^ ions into the roots of plants causes osmotic stress, which further disturbs the balance of essential nutrients within the plants resulting in ionic stress, and the superposition of these two stresses causes oxidative stress (Figure 1).

### 2.1. Osmotic Stress

The plants face osmotic stress when their roots are subjected to high salt concentrations such as Na^+^ and Cl^−^ ions. Most plants can tolerate up to 40 millimoles per liter of sodium chloride as the maximum threshold level for salt stress [11]. During the initial phase of osmotic stress, the plant’s capacity to absorb water from the soil decreases, leading to dehydration. This occurs because the osmotic pressure of the salt solution in the external soil is higher than that of the root cells (Figure 1). It causes harm to the photosynthetic machinery, resulting in the disruption of the thylakoid membrane and enzymes involved in the Calvin-Benson cycle. The disruption leads to a decrease in the levels of certain plant metabolites. This phase remains for a short interval of time causing the stomatal closure and inhibiting the shoot cell expansion [12]. Thus, osmotic stress largely affects the formation and development of plant regenerative organs such as new shoot formation. It further changes the flowering, maturity, and fruiting periods of plants [13,14]. Moreover, the excess Na^+^ ions within the plant cells damage the physiological processes, ultimately resulting in reduced seed germination and stunted seedling growth.

### 2.2. Ionic Stress

The excessive uptake of Na^+^ and Cl^−^ ions through the transpiration streams under salinity stress causes plant cytotoxicity and disrupts the nutritional balance, leading to long-term ionic stress (Figure 1). The accumulation of high levels of Na^+^ and Cl^−^ ions within the soil media instigates toxic ionic ratios such as Na^+^/K^+^, Na^+^/Ca^2+^, Ca^2+^/Mg^2+^, and Cl^−^/NO^3−^, which replaces the crucial nutrients and builds a struggling situation for the uptake of nutrients into the crops [15]. The excess of external Na^+^ ions can antagonistically affect the intracellular influx of K^+^ ions. When plants are exposed to excessive Na^+^ ions, the inhibition of K^+^ ions leads to an increase in the Na^+^/K^+^ ratio, causing K^+^ loss [16]. Furthermore, the excessive buildup of Na^+^ ions in the shoots of plants leads to a decrease in the levels of other important metal cations, such as Ca^2+^ and Mg^2+^. It detrimentally impacts the physiological and biochemical processes of the plants accelerating the senescence [17]. Moreover, in certain plant species such as citrus, Cl^−^ ions have a greater toxicity than Na^+^ ions [18]. However, the toxic threshold level of Cl^−^ ions for most plants is higher than Na^+^ ions [19].

### 2.3. Oxidative Stress

Salinity stress is accompanied by oxidative stress due to the excessive generation of reactive oxygen species (ROS), such as O^2−^, H_2_O_2_, -OH, and HO_2_ [20]. The excess ROS cannot be replaced from the plants at a rapid rate through antioxidant enzymes, causing the degradation of proteins and metabolites (Figure 1). This occurs due to the changes in the amino acid compositions, breakdown of their chains, and buildup of reaction products that are cross-linked. ROS further initiates impulsive oxidative reactions on unsaturated fatty acids and causes the formation of lipid free radicals. Thus, the plasma membrane gets destabilized by the ROS that induces peroxidation of lipids and disintegration of protein molecules, resulting in impeded integrity [21]. Eventually, under persistent conditions of salinity stress, the disruption of cell membranes and organelle stability leads to severe hindrance in nutrient biosynthesis and transport [22]. In addition, the increase in ROS inhibits the transcription mechanism and stress signal transduction that severely damages and inhibits plant growth and development.

## 3. Plants Adaptative Mechanism to Salinity Stress

**Figure 2:**
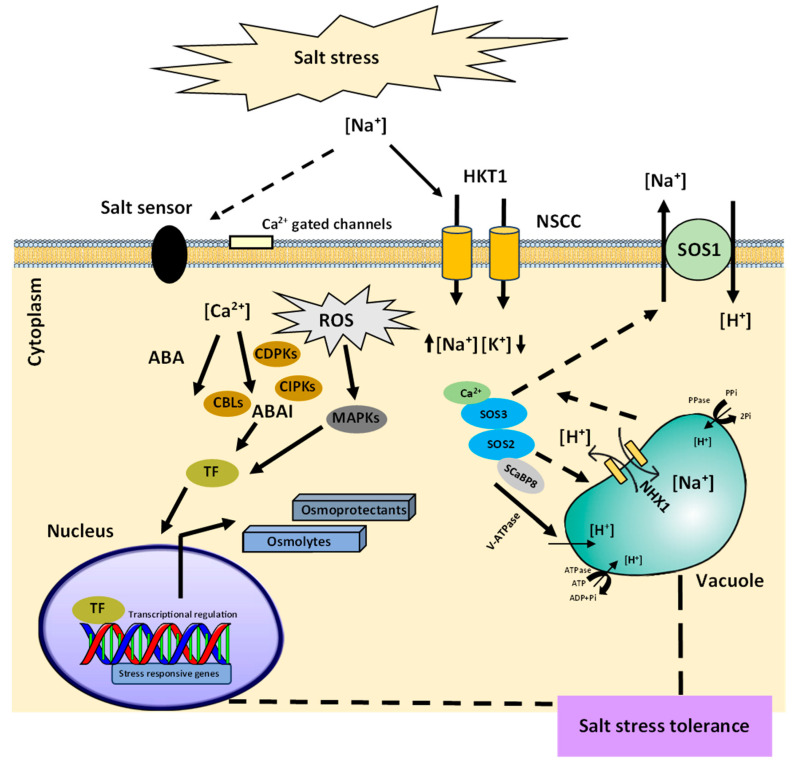
Schematic representation of plant salinity stress adaptation. Plant senses Na^+^ import intracellularly or extracellularly through both ABA-dependent and -independent signaling pathways. The regulatory pathways are induced through the initiation of various PK activities such as CDPKs, CIPKs, and CBLs. Ca^2+^ plays a major role in both the osmotic and ionic stress responses. The plants abate the ionic stress through either the exclusion of Na^+^ from the cells by SOS1 or through its sequestration into the vacuole with NHX1 transporters. The activation of transcriptional machinery increases or decreases certain stress-responsive gene expression that causes the synthesis of metabolites to counter the NaCl stress through the regulation of growth and metabolism.

Plants have developed regulatory pathways to acclimatize to salt stress. It comprises mainly signal perception, signal transduction, and responses to stress. The initial step for activating the salinity stress signaling cascade involves the recognition of stress through the plasma membrane receptors that sense physicochemical osmotic signals and chemical signals due to the excess Na^+^ and Cl^−^ ions. (Figure 2). With the identification of the salinity stress, Na^+^ ions interact with the negatively charged components of the cell wall such as rhamnogalacturonan-II (RG-II) [23]. Moreover, with the increase in Na^+^ ions within the soil, several other carriers, or channels such as high-affinity K^+^ transporters (HKTs), glutamate receptors (GLRs), aquaporins, and nonselective cation channels (NSCCs) of root epidermal cells gets involved in the transport of Na^+^ ions into the plant cells (Figure 2). This changes the mechanical tension and turgor of the cell wall and leads to the opening of stretch-activated ion channels such as Ca^2+^ channels, causing an increase in the cytoplasmic Ca^2+^ ions that act as a secondary messenger of salt stress in addition to the ROS, diacylglycerol (DAG), inositol phosphates (IPs) [24,25]. Immediately, after the stress perception, the secondary messengers trigger a set of ROS-modulated protein kinases (PKs), protein phosphatases (PPs), and TFs that induce the stress-responsive genes for the plant’s adaptation to salinity stress [26,27].

The secondary messengers initiate the downstream responses to salinity stress and cause the modulation of stress-responsive genes through abscisic acid, ABA-dependent or -independent regulation mechanism (Figure 2). The common salt stress signal transduction pathways such as the salt overly sensitive (SOS) pathway and mitogen-activated protein kinase (MAPK) cascades play essential roles in the salt stress responses [28]. The SOS pathway governs root ion homeostasis under salinity stress [29], whereas the high-osmolarity glycerol (HOG) MAPK cascade pathway controls the osmotic regulation during hyperosmotic stress [28]. In general, salt stress signal perception may activate multiple signaling pathways, which, along with crosstalk between the pathways, enable plants to adapt to salt stress.

The SOS signal transduction cascade to protect the cells from damage gets activated after the perception of a Ca^2+^ spike in the cytoplasm of root cells [30]. SOS3 encodes a myristoylated calcium-binding protein, which acts as a primary calcium sensor to sense the increase in cytosolic Ca^2+^ triggered by excess Na^+^ ions in the cytosol. Upon binding with Ca^2+^, SOS3 interacts with and activates the serine/threonine protein kinase SOS2 which is part of the SnRK3 protein kinases family (sucrose non-fermenting-1-related protein kinase-3) [31]. SOS3-like calcium-binding protein 8 (SCaBP8) has been displayed to be an alternative regulator of the activity of SOS2, which functions primarily in the Arabidopsis shoot, while SOS3 is more eminent in the roots [32]. SCaBP8 is phosphorylated by SOS2 which stabilizes the protein complex [33]. Phosphorylation of SOS3-like proteins by their interacting protein kinases is a common regulatory pathway for CBL/SCaBP–CIPK/PKS modules [34]. SOS3–SOS2 or SCaBP8–SOS2 interactions transfer SOS2 to the plasma membrane causing the activation of downstream target SOS1, a Na^+^/H^+^ antiporter. This results in the subsequent extrusion of toxic Na^+^ ions from the cytoplasm [35].

The PK, SOS, Ca^2+^, and certain phytohormones involved in this mechanism are governed by responsive genes, which are induced at the early and late stages of stress [36]. Early induced genes include TFs that are expressed swiftly after the perception of the stress, whereas the activation of late genes occurs gradually and may take several hours after the perception of stress to generate a response. However, these genes have sustained expression that modulates the required stress-responsive proteins [36]. The products of late genes amplify the initial stress signal and trigger a secondary round of signaling, which may follow the same pathways as earlier or branch off into a new signaling pathway.

These pathways can include ROS detoxification which involves the antioxidative enzymes for scavenging the toxic free radicals [37]. Salt sequestration into cell vacuoles by transporters (NHX1) is another core pathway used by plants to preserve a high cytosolic K^+^/Na^+^ ratio that regulates the toxic levels of salt ions in the cytosol for ion homeostasis (Figure 2). In addition, plants release and induce organic compounds, which are termed osmolytes or compatible solutes, stress-responsive proteins [38], and osmoprotectants [39]. Possible outputs of these compounds include the expression of genes and activation of osmolyte biosynthesis enzymes. Most of the other changes induced by salinity stress can be considered to be involved in detoxification signaling. Furthermore, the osmolytes shield the essential proteins through the exclusion of hydrophilic molecules from their hydration sphere, which inhibits or reduces their interaction with water and protects their native structures. Moreover, the plants overcome the salt stress through cell wall modifications, membrane system adjustments, cell cycle, and cell division modifications.

## 4. Phytomorphological Changes in Plants Grown under Saline Conditions

Plants have developed various mechanisms in response to salinity stress that enables them to alter their morphological traits, resulting in sustained tolerance to high salt levels. The salt-tolerant plants possess shallow roots and develop many stilt or prop roots from their aerial branches of stem for efficient anchorage in saline soils [40]. Plants grow these roots downward that enter the deep and tough strata of the soil. For instance, in *Rhizophora mucronata*, the stilt roots are strong and extensively developed, while in other species they are poorly developed such as in *Rhizophora conjugate* [41]. However, there are certain saline-tolerant plants with the absence of stilt roots. Furthermore, salt stress-tolerant plants consist of a large number of adventitious root buttresses that sprout from their basal parts, which provides them sufficient support in salt-affected soils [42]. In the coastal areas, the saline soils are poorly aerated, containing a very small proportion of oxygen due to water logging. To overcome the lack of soil aeration, the plants develop negative geotropic roots, known as pneumatophores or breathing roots [43]. The pneumatophores are developed from the underground roots and projected towards the air well above the surface of soil and water, which appears as peg-like structures with pointed tips [43]. These roots surface possess numerous lenticels or pneumathodes and prominent aerenchyma that encloses the large air cavities internally. Lenticles are used for the gaseous exchanges in these roots and the aerenchyma is involved in the conduction of air down to the roots that are submerged. For example, in *Bruguiera*, the horizontal roots develop above the surface of the soil and then again bend down and enter deep into the soil forming knee-like structures [44]. The gaseous exchange is further facilitated by the pores in the aerial surface of the roots. However, in certain species such as *Rhizophora*, pneumatophores do not develop, due to which the respiratory activity under saline conditions is taken up by the upper aerial parts of descending stilt roots. Another key factor in coping with salinity stress is the plasticity of roots. The root morphological plasticity can restrict the buildup of salt ions in roots to allow the uptake of water under saline soils [45]. It was found that at lower salt stress concentrations of 5 g L^−1^ NaCl, an abundance of root hairs was induced, but it gradually declined under greater salt concentrations of 10 and 15 g L^−1^ NaCl in *Bacopa monniera* [46]. Moreover, in wheat plants under salinity stress, both the length and density of root per unit surface area were less than 25% and 40% in contrast to the hydroponically grown plant genotypes [47].

Stems of several salt-tolerant plants develop succulence, which is commonly found in *Salicornia herbacia* and *Suaeda maritima*. The succulence formation depends on the ratio of absorbed to free ions in the plant cells rather than the amount of absolute NaCl present [48]. Succulence growth is stimulated with the increase in free salt ions above the threshold level within the plants. In salt-tolerant citrus species, *Cleopatra mandarin*, an increase in the succulence of leaves was found under the salinity stress [49]. According to Qi and Zhang, cell division gets inhibited during the salinity stress that promotes cell elongation [48]. This causes a decrease in the number of cells and an increase in the cell size, which commonly occurs in succulents. Fradera-Soler et al. (2022) stated that succulence is directly associated with the plant’s salt tolerance and the degree of their development can indicate the capability of plants to survive in highly saline habitats [50]. The temperate saline-tolerant plants are herbaceous, but the tropical ones are largely bushy and display dense cymose branching. Nevertheless, there are salt-tolerant plants such as the submerged marine angiosperms that do not become succulent.

The leaves in most salt-tolerant plants are generally smaller in size, thick, entire, and succulent, with a glassy appearance [51]. In *Cleopatra mandarin*, salt-tolerant citrus species, an increase in the leaf thickness with a lower area/volume ratio of mesophyll cells was observed under salinity stress [49]. Similarly, under high salt stress conditions, increased leaf thickness was observed in *Lawsonia inermis* plants [52]. Furthermore, the coastal saline-tolerant plant leaves display an additional mode of adaptation to their environment. The leaves of these plants are densely covered with trichomes [53]. In submerged marine plants, the leaves are thin with a poorly developed vascular system frequently having a green epidermis that is adapted to uptaking water and nutrients directly from the medium [54]. Saline-tolerant plants develop lightweight fruits and seeds with fruit walls having several air chambers for fruit dispersal [55]. In addition to these morphological adaptations, several salt-tolerant plants undergo physiological and biochemical adaptation mechanisms that are governed by a network of stress-responsive genes controlled by the TFs.

## 5. Regulatory Network of TFs in Plants Response to Salinity Stress

**Figure 3:**
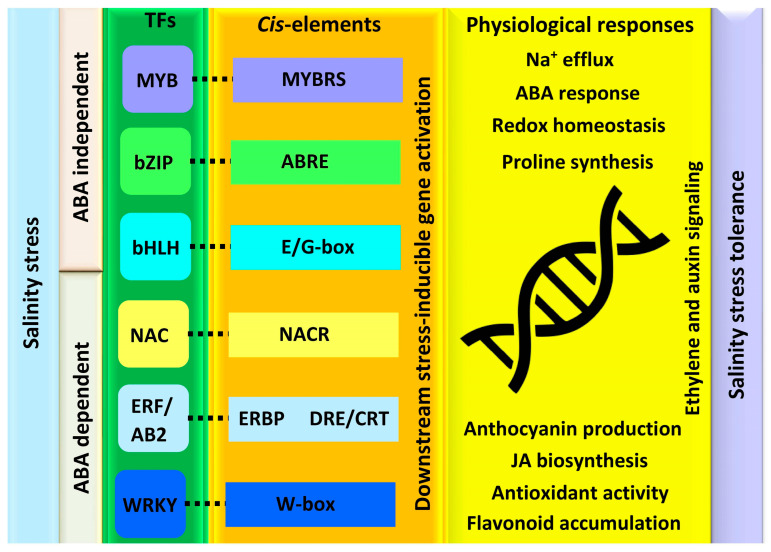
Overview of transcriptional modulation of salt stress in plants. Both the ABA-dependent and -independent pathway initiates the transcriptional regulatory networks. Salt stress signaling prompts transcription factors to bind to their corresponding cis-regulatory elements, which then triggers the expression of genes required for various physiological responses to cope with salinity stress.

Several plant biochemical alterations during salinity stress occur due to the transcriptional changes that alter the plant’s growth and developmental processes. TFs are the core elements that control the whole-plant transcriptional responses as regulatory molecular switches of the genes [56]. These are instigated by ABA-dependent and -independent pathways that function either individually or through cross-linking with other TFs (Figure 3). They induce the expression of functional genes and initiate different salt tolerance phenomena in plants [8]. Based on the genome-wide analysis, various TFs belonging to different families, such as myeloblastosis (MYB), basic region/leucine zipper motif (bZIP), basic helix-loop-helix (bHLH), NAC, ethylene responsive factor (ERF/AP2), and WRKY have been studied for salt stress tolerance [57].

### 5.1. MYB Transcriptional Regulators

MYB is one of the largest groups of TFs that are involved in most of the plant’s hormone signal transduction. These TFs are determined by a highly conserved MYB domain located at the N-terminus that facilitates the DNA binding to the cis-element MYBRS (Figure 3) [58]. MYB transcription factors are classified into four categories based on the repetition of the MYB domain. These groups are 1R-MYB, R2R3-MYB, R1R2R3-MYB, and 4R-MYB [59]. *MYB* genes are expansively involved in regulating the plant salinity stress tolerance and several of their downstream targets have been identified. It was shown in a study that under salt stress conditions, the overexpression of *Arabidopsis thaliana MYB49* TF activated several differentially expressed genes (DEGs) including *MYB41*, *ASFT*, *FACT*, and *CTP86B1*. Transcriptome analysis displayed that several of these DEGs belonged to the category of cutin, suberin, and wax biosynthesis which enhances salinity stress tolerance. In addition, the overexpression of *Oryza sativa*, *OsMYB2* stimulated the formation of osmolytes such as proline, by increasing the expression of genes involved in proline synthesis and transport. Furthermore, it has led to enhanced expression of several genes such as *OsLEA3*, *OsRab16A*, and *OsDREB2A*.This process helped to prevent the risk of oxidative damage caused by the excessive accumulation of H_2_O_2_ and MDA content in cells when exposed to high levels of salt stress [60]. Similarly, in *A. thaliana* the overexpression of *MYB12* during salinity stress activated the flavonoid, ABA, and proline biosynthesis genes that lead to enhanced tolerance [61]. Moreover, the SUMOylation of *MYB30* by SIZ1 improved the salt tolerance of Arabidopsis by maintaining cellular redox homeostasis by upregulating the expression of the *AOX1a* gene [62]. The overexpression of several TFs such as the *MYB47*, *MYB15*, and *MYB52* conferred the tolerance to salinity stress by regulating the plant defense system and decreasing the overproduction of ROS [63]. In *A. thaliana*, *AtMYB2* and *AtMYB44* regulated the salinity stress at the mRNA level, and it was found that the overexpressed lines were more tolerant to salt stress [64,65,66]. A comprehensive wide investigation of the peanut, *Arachis hypogaea* genome indicated that the expression of *AhMYB1*, *AhMYB2*, *AhMYB6*, *AhMYB6*, and R2R3-MYB was induced under salinity stress [61,67].

### 5.2. bZIP Transcriptional Regulators

bZIP is another group of essential TFs that regulate plant developmental processes and abiotic stress tolerance. They possess a sequence-specific DNA binding highly preserved domain that is basic in nature. bZIP TFs further have an adjacent heptad leucine repeat domain, which is commonly known as the leucine zipper dimerization motif [68]. The heptad repeats of leucine located at the C-terminus contain nine different amino acid residues that promote the formation of an amphipathic helix [69]. Plant bZIP TFs have a strong affinity for binding to ACGT core DNA sequences, which include G-box (-CACGTG-), C-box (-GACGTC-), A-box (-TACGTA-), PB-like (-TGAAAA-), ABRE (ABA-responsive element, -CCACGTGG-), and GLM (GCN4-like motif, -GTGAGTCAT-) [70]. These motifs are present in the promoter regions of different genes that signal the occurrence of stress (Figure 3). The bZIP TFs massively contribute to various physiological processes, such as maturation and germination of seeds, sensing of light for photomorphogenesis, responding to adverse environmental stress conditions, and plant senescence [71]. The overexpression of *Solanum lycopersicum SIbZIP38* and *O. sativa OsbZIP42* improved their salt stress tolerance [70]. *S. lycopersicum SIbZIP1* overexpression lines were found to be more sensitive to drought and salinity stress, which induced high MDA content and low proline and chlorophyll content [72]. This further downregulated the transcription levels of ABA biosynthesis genes in response to salt stress, whereas *O. sativa OsbZIP42* and *ZEP252* overexpressed lines led to enhanced tolerance to abiotic stress by exhibiting a rapidly induced expression of the ABA-responsive *LEA3* and *Rab16* genes that largely led to the activation of the ABA signaling pathway [73,74]. Recent studies indicated that wheat bZIP TFs, *TabZIP60*, and *TaZIP14-B* significantly enhanced *A. thaliana* salinity, drought, and freezing stress tolerance [75]. It was found that *TabZIP60* binds to the ABA-responsive cis-element and improves abiotic stress tolerance. These findings demonstrated that bZIP and ABA have strong interconnection during salt stress tolerance of plants. In addition, overexpression of *Capsicum annum* bZIP TF, *CabZIP25* in Arabidopsis improved its salt stress tolerance increasing the germination rate, fresh weight, root lengths, and chlorophyll content in contrast to the wild-type Arabidopsis plants [76].

### 5.3. bHLH Transcriptional Regulators

bHLH TF is the second largest group of TFs, which is named due to its highly preserved alkaline/helix-loop-helix domain [77]. It consists of approximately sixty conserved residues of amino acid that are composed of two preserved motifs, including a basic region and a helix-loop-helix region (HLH region). The basic region of bHLH is involved in the binding of DNA to the E-box (-CANNTG-) or G-box (-CACGTG-) motifs present in their target genes (Figure 3). While the region of HLH consists of two alpha helices comprised of residues that are hydrophobic, which are needed in the dimerization and subsequent regulation of target gene expression involved in different pathways of stress signaling. Based on their DNA binding function and phylogenetic relationship, the bHLH sub-members are classified into six different groups [78]. In general, Group A specifically binds to the core sequences of E-box, while G-box binds to Group B, and Group C binds to specific sequences either -ACTTG- or -GCGTG- [79]. Whereas the basic region is not present in Group D and is primarily involved in the heterodimerization with other molecules of bHLH proteins [80]. Moreover, Group E bHLH TFs bind to the sequence of CACGNG, and the members of Group F to certain target sequences of DNA [81].

Various *bHLH* genes are involved in improving the salinity stress tolerance of plants. The bHLH TF was identified in Arabidopsis calcium-binding NaCl-inducible gene 1, *AtNIG1* that conferred an enhanced salt tolerance with its overexpression through osmotic balance and proline synthesis than its corresponding knockout mutants [82]. Likewise, the expressions of *AtbHLH122* and *AtbHLH92* provided an increased salt tolerance in *A. thaliana* [83,84]. Improved salinity stress tolerance was further reported in *A. thaliana* that were heterologously expressing bHLH TF of *Myrothamnus flabellifolius*, *MfbHLH38* [85]. Furthermore, the bHLH regulates the ion transport during the salt stress tolerance, by controlling the Na^+^/H^+^ antiporter NHX. *AtMYC2* and *AtbHLH122* TFs were observed to act as upstream regulators of Arabidopsis *NHX* genes, *AtNHX1* and *AtNHX6*, that confer Arabidopsis salt stress tolerance [86]. Salinity stress tolerance was improved in transgenic *A. thaliana* plants overexpressing the *Oryza rufipogon bHLH* gene, *OrbHLH001* [87], whereas in rice cultivars, the overexpression of inward-rectifying K^+^ channel (OsAKT1), *OrbHLH001* maintained the balance of ions under salt stress [88]. In addition, maize bHLH, and *ZmbHLH55* enhanced the salt stress tolerance through the increased ascorbic acid accumulation by direct regulation of the genes included in the biosynthesis of ascorbic acid [89]. It was found that the *Zmbhlh55* mutant positively regulated the *ZmPGI2*, *ZmGME1*, and *ZmGLDH* gene expressions, but negatively regulated the expression of *ZmGMP1* and *ZmGGP*. These studies indicated the various regulatory networks through which the members of the *bHLH* gene group enhance plants’ salinity stress tolerance. However, under salinity stress, a series of secondary stresses will occur and the specific function of bHLH TFs in regulating ions and metabolites balance needs to be investigated in plants.

### 5.4. NAC Transcriptional Regulators

NAC is an important TF that is determined by a highly preserved DNA-binding NAC domain in the N-terminal site, and its C-terminal transcription regulatory site consists of a transmembrane domain that activates or represses the transcription of genes under abiotic stress conditions [90]. These TFs bind to the cis-element of *NACR* and are involved in the ABA-dependent or -independent pathways for the induction and regulation of stress tolerance (Figure 3) [91]. It was observed that *A. thaliana RD26*, which encodes for NAC protein acts as a transcriptional activator for the ABA signaling pathway to improve the salinity stress tolerance [92]. Similarly, NAC-type TFs *AtNAC2* and *NAC57* enhanced the salt stress tolerance of *A. thaliana* through the ethylene and auxin signaling pathway activation [93]. Furthermore, in *S. lycopersicum*, *SINAC4* reacts to salt stress by regulating the methylation of JA and downregulating the biosynthesis of ABA [94]. This indicated that *SINAC4* is involved in the ABA-independent signaling pathway to regulate salinity stress tolerance. Moreover, in *A. thaliana*, overexpression of the *NAC* gene in transgenic rice displayed improved salt tolerance and insensitivity to ABA that elevated the transcription of *OsLEA3*, *OsSalT1*, and *OsPM1* stress-associated genes [95].

### 5.5. ERF/AP2 Transcriptional Regulators

Plant abiotic stress tolerance is mediated by plant hormones such as ethylene. In relation to this, ERF TF regulates the signaling and biosynthesis pathway of ethylene for improving salt stress tolerance. There are plant-specific TFs known as ERFBP or AP2 family that consists of a highly conserved DNA binding domain, ERF/AP2. These TFs are classified into different subcategories such as dehydration-responsive element binding protein (DRE), ERF, and AP2, which are linked with ABI3 “Abscisic acid insensitive3”/VP1 “Viviparous1” [96]. It binds to the ERBP or DRE/C repeat element and to the GCC box in the downstream gene promoter regions (Figure 3) [97]. Various *ERF* genes have been found to modulate salt stress tolerance. In wheat, *TaERF3* overexpression enhanced the salt stress tolerance by regulating the stress-responsive genes and enhancing the expression of *LEA3*, *DHN*, *BG3*, *RAB18*, *Chit1*, *SDR*, *TIP2*, *POX2*, *OxOx2*, and *GST6* genes in Yangmai 12 lines. The overexpression of these genes resulted in an increase in the accumulation of both proline and chlorophyll and decreased the H_2_O_2_ content and stomatal conductance [98]. Furthermore, *A. thaliana* lines with overexpressed *ERF1* had improved salt tolerance through the activation of antioxidant enzyme activities and stabilization of ROS production [99]. It was indicated that ERF1 upregulated certain sets of genes such as *LEA4-*5, *RD20*, *RD29B*, *COR47*, *HSP17.6A*, that played a role in the salt stress tolerance by binding to stress specific GCC or DRE/CRT motifs and activating genes associated with ethylene, jasmonic acid (JA), and abscisic acid (ABA) [99]. It further upregulated the expression of *P5CS1*, which encodes for the proline synthesis under abiotic stress. Dubouzet et al. (2003) described five types of *DREBs*, *DREB1A*, *DREBB*, *DREBC*, *DREBD*, and *DREB2A* in *O. sativa* that were overexpressed in *A. thaliana*, which activated several stress-inducible genes and elevated the tolerance level of plants to salinity, cold, and drought [100]. In addition, ERF/AP2 TFs play a crucial role in regulating various stress responses, such as the release of plant hormones and the proliferation of cells for reproductive and vegetative growth in plants [101].

### 5.6. WRKY Transcriptional Regulators

WRKY is another group of TFs that play a crucial role in several plant processes such as seed development, growth, leaf senescence, and responses to abiotic stresses [8]. WRKY TFs consist of either one or two domains that are preserved, each containing approximately sixty residues of amino acid. The WRKY transcription factors have a preserved WRKYGQK domain at the N-terminal and a C2HC/C2H2 domain at the C-terminal [102]. The W-box cis-elements with the core sequence of TTGACC/T interact with the WRKY domains, which are in the promoter region of specific target genes (Figure 3) [103]. These TFs are classified into three main groups based on their number of domains. Group one of WRKY TFs contains two WRKY domains and one C2H2 zinc-finger motif. On the other hand, groups two and three have one WRKY domain with a C2HC and C2H2 zinc-finger motif, respectively [104].

In the plant’s salinity stress tolerance, several *WRKY* candidate genes have been reported. The overexpression of chrysanthemum, *Dendranthema grandiflora* TFs *DgWRK1*, *DgWRK2*, *DgWRKY3*, and *DgWRK5* in transgenic chrysanthemum plants enhanced their salinity stress tolerance through upregulation of several stress-responsive genes such as *DgCAT*, *DgNCED3A*, *DgNCED3B*, *DgCuZnSOD*, *DgP5CS*, *DgCSD1*, and *DgCSD2*, which increased antioxidant enzyme activities and decreased ROS accumulation [105,106]. Furthermore, the overexpression of *O. sativa* TFs, *OsWRKY45*, and *OsWRKY72* improved the salt stress tolerance by inducing the genes involved in the regulation of ABA and auxin signaling pathways [107,108]. It was found that three auxin-associated genes *AUX1*, *AXR1*, and *BUD1* were highly altered in rosette leaves and inflorescences of 35S-OsWRKY72 plants in contrast to the control Arabidopsis, and in 35S-OsWRKY72 seedlings, two ABA-related genes *ABA2* and *ABI4* were instigated. Similarly, in *Zea mays*, the modulation of TFs, *ZmWRKY23*, *ZmWRKY48*, *ZmWRKY58*, and *ZmWRKY86* improved the tolerance to salinity through the activation of ABA signaling pathway that stimulated the plant defense mechanism [109,110]. In another study, the wheat TF, *TaWRKY10* largely enhanced the tobacco salt stress tolerance by regulating the expression of several stress tolerance-related genes such as *NtERD10C*, *NtSPSA*, and *NtGPX*. The overexpression of these genes enhanced the osmotic balance, facilitated antioxidant enzyme activities, and minimized ROS accumulation in tobacco [111]. *BcWRKY46* from *Brassica campestris* ssp. chinensis improved tolerance to different abiotic stresses including salinity stress in tobacco plants [112]. The Arabidopsis *WRKY33* TF is shown to target the downstream genes that respond to salt stress, detoxifying ROS, regulating lipoxygenase (LOX1), peroxidases (POD), and glutathione-S-transferase (GSTU11) [113]. Zheng et al. (2013) found that under salt stress, *Tamarix hispida ThWRKY4* modulated the cellular protective mechanism toward the toxic levels of ROS [114].

### 5.7. MicroRNA (miRNA) as Transcriptional Regulators

miRNAs are non-coding single strand small RNAs that have 21–24 nucleotides [115]. They are essential regulators of gene expression in plants and control the transcript of target genes involved in various plant physiological processes and response to adverse abiotic stress tolerance. It has been reported that overexpression of a miR393-resistant *TIR1* gene (*mTIR1*) in Arabidopsis enhanced the salt stress tolerance by improving the germination rate, root elongation, chlorophyll accumulation, and prevented the water loss [116]. The transformed Arabidopsis plants accumulated more proline and anthocyanin and displayed upregulation in the expression of certain salt stress-related genes such as *CMO* (*At4g29890*), *ALDH10A8* (*At1g74920*), *ALDH10A9* (*At3g48170*), *SOS1* (*At2g01980*), *AVP1* (*At1g15690*), and *NHX1* (*At5g27150*) that reduced the excess of sodium content [116]. Furthermore, it was found that *miRNA396c* overexpression enhanced the salinity stress tolerance of cotton plants through the regulation of water retention, chlorophyll accumulation, cell membrane Na^+^/H^+^ levels, antioxidant enzymes, and TFs [117]. The overexpression of *miR395c* and *miR395e* improved the seed germination of transformed Arabidopsis plants under salt stress, by targeting *APS1*, *APS3*, and *SULTR2* genes [118]. In another study, it was shown that *miR172c* is largely induced by salt stress in soybean. *miR172c* overexpression and knockdown substantially enhanced and decreased root sensitivity to salt stress, respectively, by altering the expression of the *NNC1* gene [119]. In addition, the overexpression of *miR408* in Arabidopsis led to enhanced tolerance to salinity stress. This occurred due to the cellular antioxidant capacity improvement as manifested by reduced levels of ROS by inducing the expression of *CSD1*, *CSD2*, *GST-U25*, *CCS1*, and *SAP12* [120]. Moreover, the overexpression of a wheat miRNA, *TaemiR408* upregulated the transcripts of abscisic acid (ABA) receptor and SnRK2 protein-encoding genes, *NtPYL2* and *NtSAPK3*, which resulted in an enhanced salt stress tolerance [121].

Most of the stress regulatory mechanisms have been examined in model crops. However, it is essential to evaluate the CWRs possessing valuable genes with immense roles in crop improvement and adaptation to salinity stress. Thus, the CWRs’ genetic resources need to be fully exploited for their beneficial traits associated with salt stress tolerance.

## 6. Crop Wild Relatives (CWRs): Valuable Repository for Salinity Stress Tolerance

Contemporary agricultural systems heavily rely on a small proportion of highly productive crops. Only twenty plant species are cultivated to meet ninety percent of the world’s food calorie requirements. Among these, three crops such as wheat, rice, and maize are supplying about 60% of the total food [122]. More than 10,000 years ago, the domestication of these food crops occurred from their wild relatives that were distributed across a broad range of habitats, including salt marshes. The genetic traits largely contributed to the CWRs’ ability to thrive under salinity. During the course period, there was a significant transformation in the performance and genetic structure of the CWRs [123]. By selectively breeding a small number of wild relatives with beneficial characteristics, such as non-brittle rachis, compact plant stature, and loss of germination inhibition, landraces with improved growth performance were created. However, in subsequent generations, this process led to a gradual reduction in genetic diversity [124]. The shrinkage in the genetic diversity of crops has been further intensified by contemporary techniques of breeding plants, which focus on producing improved yielding cultivars by crossing landraces that are productive and ignoring the wild relatives that possess larger genetic diversity but have poor agronomic characteristics [125]. Such narrowing of genetic diversity has been experimentally confirmed [126]. Moreover, the shift of farmers from cultivating local crop varieties and landraces to genetically uniform and high-yielding varieties has led to a loss of about 75% of genetic diversity in crops [122].

Importantly, as present techniques of plant breeding tend to be performed in optimized agricultural settings, the genetic elements of abiotic stress tolerance including salinity, are frequently amongst the lost fractions. Hence, the attempts to find genes that provide tolerance to the salinity stress within the commercially used varieties have produced only restrictive outcomes. Therefore, the CWRs are essential resources for genetic elements of salinity stress tolerance that can be utilized in contemporary agricultural breeding [127]. For example, *Hordeum marinum*, sea barley grass is classified as a wild halophyte, whereas other species of Hordeum are considered as glycophytes. A physiological analysis revealed that sea barley grass (H559) demonstrated greater salinity tolerance compared to the barley genotypes XZ113 and Golden Promise [128]. Another study found that certain accessions of Tibetan wild barley exhibited superior salinity tolerance in contrast to the well-established saline-tolerant barley cultivar CM72 [129]. *Oryza coarctata (Porteresia coarctata)* is a wild halophytic rice found in the coastal environments, which possessed significant tolerance to both the salinity stress and submergence [130]. Similarly, several CWRs were identified to be salt stress tolerant (Table 1).

The broad variation in the salt stress tolerance quantitative trait locus (QTL), TFs, and genes of CWRs can serve as valuable sources for the enhancement of the cultivated species However, this genetic diversity needs to be examined further for the identification of essential molecular pathways and the utilization of various elements involved in them.

## 7. Modern Molecular Technological Advancements for the Identification and Utilization of the Genetic Diversity of CWRs for Salt Stress Tolerance

It is essential to deploy CWRs for the development of salinity stress tolerance due to the shrinkage of the genetic diversity in modern crop cultivars. It has mainly occurred because of the focus of current breeding and genetic approaches to produce high-yielding elite cultivars through the crossing of productive landraces and ignoring wild relatives [125]. Such narrowing of genetic diversity has been experimentally confirmed [126]. The lack of genetic diversity among the modern elite cultivars can be complemented by several varied genetic sources among the wild relatives of crop [122]. Genetic diversity developed artificially through mutagenesis has proven to be an essential resource for different species of crops, as variants can be produced directly from the commercially available germplasm [144,145]. However, generating salt-tolerant variants through artificial means is limited because salinity stress tolerance is primarily the result of the combined effects of multiple mechanisms. Salinity stress tolerance is more likely to evolve through the natural selection of crops that are exposed to hostile conditions of the environment, as found with wild relatives and landraces [146]. Despite showing large capabilities in salt stress tolerance, the crucial genetic resources from wild crop relatives have remained comparatively untapped because of the relative complexity of their germplasms [147]. In addition, it can be difficult to precisely characterize widely diverse and undomesticated crop panels through phenotyping. The restrictive genetic resources from the wild relative germplasms along with challenges in efficiently transferring advantageous alleles into elite crop varieties have proven to be a significant hindrance [147]. Nonetheless, modern technological advances in DNA sequencing and phenotyping have enabled the identification of genetic resources from several saline-tolerant CWRs.

The rapid advancement in DNA sequencing and genome assembly creation over the last ten years has facilitated the identification of potential salt tolerance alleles, genes, and single nucleotide polymorphisms (SNPs) in CWRs [148]. Most of this progress is due to the decreasing cost of short-read sequencing technologies. However, developing long-read, real-time, linked-read, and single-molecule sequencing methods have further played a significant role in overcoming the inherent challenges posed by plant genomes, including their bigger size, frequent ploidy complexity, and high repeat content [148]. By producing high-quality reference sequences, researchers are able to analyze the genomes of wild crop relatives and identify the genetic variations that contribute to species-specific salt stress tolerant traits. For example, genes responsible for drought and salinity stress tolerance have been pinpointed in wild tomato species such as *S. pennellii* and *S. pimpinellifolium*, respectively [149,150]. Moreover, quinoa (*Chenopodium quinoa*), a pseudo-cereal, has recently become popular in developed countries due to its nutritional potential and a significant amount of genetic and phenotypic diversity [151]. Certain quinoa accessions have even demonstrated high salt stress tolerance [152]. The study of genomes of two elite quinoa, together with molecular data from an increasing accession and wild relatives, provided the crucial potential for the identification of the genetic elements involved in the elevated salinity stress tolerance [153]. Interestingly, efforts are currently underway to domesticate these wild crop relatives that are already naturally tolerant to salt stress and to transfer their tolerance traits into major crops.

Global gene banks have further gathered about two million distinct plant accessions, with a significant proportion consisting of landraces and CWRs. The possibility of utilizing the germplasm repositories of these crops as a resource of natural diversity for the identification of salt stress tolerance has been widely studied [151]. Genetic diversity investigations have been carried out on various collections of crop germplasm, including wheat [154,155], rice [156,157], maize [158,159], barley [160], and tomato [161]. In addition, different studies utilizing the forward genetics technique have already displayed the practical importance of using varied germplasm by identifying loci linked to several measures of salinity stress tolerance in well-established crops, such as soybean, rapeseed, and alfalfa [162,163,164].

The next-generation sequencing (NGS) of distinct plant accession in global gene banks will lead to the development of de novo genome assembly that will facilitate the compilation of pangenomes. Several CWR and landrace genomes of various crop species have been subjected to de novo genome assembly and resequencing (Table 2). It provided a more comprehensive insight into structural and genetic variations across several plant genotypes [165]. High-quality whole-genome assemblies of wild relatives of chickpeas [166], and 19 wild rice species [167,168,169] were further investigated. At present, studies are being conducted with the objective of capturing the whole genetic material of a plant species. A major role of structural variations in crop domestication has been evidenced from pangenome studies of different crops. For instance, a pangenome analysis of the *O. sativa*-*O. rufipogon* using de novo genome assemblies of sixty-six diverse accessions revealed a significant presence–absence variation in genes that govern their time of flowering and hull color [170]. This shows that pangenome analysis provides larger opportunities for the identification of genetic diversity that may have been lost, targeted against, or merely considered within the gene pool of the crop domestication process. A pangenome study was performed on 725 tomato accessions, which identified a rare allele for the tomato lipoxygenase gene, TomLoxC. Specifically, there was a substitution of approximately 4 kb in the promoter region of this gene [171]. The investigation further reported 4873 genes that were not present in the reference genome of the tomato plant Heinz 1706. Likewise, a pangenome study including 1961 accessions of cotton plants revealed several genes that were absent in the *Gossypium hirsutum*, TM-1, and *Gossypium barbadense* used as reference genomes, respectively [172]. These genes are described to be belonging to the dispensable portion of the species genome, because they can be either present or absent without affecting the viability of the organism.

To develop an efficient illustration of wide-ranging diversity among CWR genomes, Khan et al. (2020) suggested the broadening of the current pangenome approach to a genus-level strategy for constructing a super-pangenome [190]. The creation of a super-pangenome involves the de novo assembly of representative accessions and the resequencing of available accessions within a particular species. A coherent graph will be developed based on this super-pangenome that combines multiple species-specific pangenomes. It will provide insight into the vast genomic diversity of crops including their wild relatives [190]. Otherwise, the lack of advanced genome-wide techniques makes it difficult to access and understand the wide variety of CWRs. Thus, constructing a pangenome at the genus level can potentially reveal untapped genetic variation that is currently hidden within a particular CWR. This would enable the examination of the dispensable portion of a species’ genome, which could greatly enhance our understanding of crop adaptation and genomic evolution. Moreover, the accessibility to the reference genomes, pangenomes, and genotypic resources from wild crop relatives, alongside the growing refinements in biotechnological tools such as transcriptomics, will reveal the underlying mechanisms and the genetic basis of salt tolerance in CWRs. This will offer the needed progress for finding novel genotype and phenotype relationships that are involved in enhancing crop salinity stress tolerance.

## 8. Transcriptomic Tools for Investigating Salt Tolerance Regulators in CWRs

The current developments in molecular plant science have broadened the collective understanding of plant salt stress tolerance. Transcriptomics has largely become a potent method for comprehending how genes react differently to salinity stress. It is utilized to study the sequences and functions of the non-coding and coding RNAs in plants [191]. Analysis of gene expression using transcriptomics of tissue facilitated the annotation of genes that were previously unannotated from the CWRs for salinity stress tolerance [191,192]. Several transcriptomics investigations have demonstrated the quantity of clustered transcript reads, as well as the downregulated and upregulated genes in crops exposed to salinity stress. Gene identification techniques can be performed based on the analysis of expressions, such as subtractive hybridization (SSH) [193], serial analysis of gene expression (SAGE) [194], expressed sequence tags (ESTs) [195], and massively parallel sequencing (MPSS). In the contemporary transcriptomic approaches, microarray and RNA sequencing (RNA-Seq) are the primary methods used for transcript analysis.

Microarrays developed in the 1990s, with the initial results released in 1995 as a means of analyzing gene expression. A single microarray chip has the capacity to analyze a sample with thousands of genes at once. For instance, the 22-k Barley1 gene chip [196] and the Model Organism Barley Gene Expression Microarray, 4 × 4-k, were initially utilized. At the primary stages, it was only possible to run one sample per chip. With technological advancements, multi-sample gene chips were developed. Furthermore, transcriptomic analysis using microarrays has been performed for several model plants under salt stress conditions, which includes rice [197], wheat [198], maize [199], potato [200], sorghum [201], barley [202], and Arabidopsis [203]. However, few of the CWRs were studied using this technique, which needs to be utilized for broadening the identification of salt stress tolerant transcripts in these crops.

RNA-Seq on the other hand is a high-throughput sequencing technique that involves the sequencing of entire RNA in a sample. It has the capacity to replace microarray analysis [204]. RNA-Seq is much more efficient than microarrays, primarily due to its ability to identify noncoding RNA, alternative splice junctions, allele-specific expression, and novel transcripts, even when they are present in low abundance. This technique does not need prior information related to annotations or assembly sequences. RNA-Seq is not biased, which can be a limitation of probe hybridization in microarray experiments [205]. Comparisons of gene expression profiles from the identical samples using Illumina and microarray analysis indicated that RNA-Seq produced more robust results [204]. In this study, it was observed that RNA-Seq detected transcripts with extremely low abundances, DEGs, and identified new transcripts and sequence variants in contrast to the microarray analysis. Several transcriptome investigations have been performed for salinity stress tolerance in various model plants and CWRs. These include pearl millet [206], *Rosa chinensis* [207], rice [204], and wild barley (*H. spontaneum*) leaves under salinity stress [208].

It is necessary to use transcriptomics to examine the crop species for which their genomes are not sequenced [209]. NGS techniques have simplified the design and use of RNA-Seq technologies and de novo transcriptome assembly [210]. Transcriptome analysis through de novo assembly further reveals different TFs, genetic regulators, or novel alleles that play a crucial role in responding to stress conditions [210]. It can display the differences in allelic expression of genes, including orthologues and paralogues in the study of polyploid genomes, which are essential for understanding the genomic basis of stress tolerance in CWRs [182].

## 9. Transcriptomic Profiling Approach of Wild Crop Relatives Salinity Stress Tolerance

Wild relatives of crops generally have a varied pool of genes and exhibit higher genetic variation when contrasted to the domesticated species [139]. In relation to this, the transcriptomic profiling of CWRs has revealed numerous salt stress-responsive genes [211]. For instance, to identify high salt-tolerant genes, transcriptome sequencing was performed on a wild relative of halophytic potato species, *Ipomoea imperati* [139]. A comparison was made between the transcriptome profiles of *I. imperati* under normal and salt stress conditions to annotate the stress-responsive mechanism and the related candidate genes that were involved in this process. De novo assembly initiated 67,911 transcripts and elucidated 39,902 putative genes [139]. Among these, 220 and 936 salt-tolerant genes were identified in the roots and leaves, respectively. Various salt-responsive genes were found in *I. imperati* under salt stress conditions such as *PP2C* and *SnRKs* which were critical elements of the ABA-signaling pathway, and *EIN2* and *EIN3* genes that were involved in the ethylene signaling pathway. Furthermore, receptor-like kinases (*RLK*) such as *HAIKU2* were upregulated to decrease the deleterious impact of salinity stress.

De novo transcriptome investigation was performed to detect salt stress-responsive genes in *S. chilense*, wild tomato, which can endure extreme salinization [210]. Using the technique of RNA-Seq, a comparative expression of genes from the wild and cultivated lines was conducted and 386 million clean reads were obtained. From the de novo assembly, a total of 514,747 unigenes were identified, and among these, 265,158 were found to be expressed differentially. Furthermore, 134,566 DEGs were upregulated under the salinity condition that played a role in stress signaling pathways such as ABA, auxin, gibberellin (GA), ethylene, and cytokinin (CK). In addition, novel tolerant genes encoding TFs, osmotic regulators, transporters, homeostasis maintainers, ROS scavengers, arginine, and proline metabolites were induced in the wild lines in contrast to the cultivated tomatoes [210]. In another study, a de novo transcriptome analysis was conducted on *G. aridum*, resulting in the assembly of 98,989 unigenes from 41.5 million obtained transcripts. Several DEGs involved in stress signaling, transporting, and hormone-stimulating pathways were both up- and downregulated under different salt stress conditions. The genes that governed the activity of transporters and protein kinases were dominantly upregulated providing salt stress adaptation to *G. aridum* [212]. In addition, Wei et al. (2017) used the transcriptomic profiling of *Gossypium klotzschianum*, wild cotton roots, and leaves to investigate the expression patterns of genes and the fluctuation of plant hormones under salinity stress [213]. RNA-Seq analysis recovered 37,278 unigenes and detected 14,000 DEGs in the roots and leaves that were involved in the salinity stress tolerance. The study revealed that *PSY*, *BCH*, *NCED*, and *CYP707A* genes of wild cotton significantly differed in expression under salinity stress. Furthermore, in wild cotton plants, *SAM*, *ACC*, *ACS*, and *ACO* genes were highly upregulated after exposure to salt stress. The analysis of gene functions indicated that certain identified genes were involved in ion homeostasis, signal transduction, and SOS pathway under salt stress. The transcriptomic data obtained from this study provided a comprehensive understanding of the mechanism underlying salt stress tolerance in cotton plants. The identified genes serve as a valuable genetic resource for improving cotton plant growth under abiotic stress conditions.

Wu et al. (2017) performed a de novo-wide profiling of transcriptomes from the *Fagopyrum tataricum*, commonly known as tartary buckwheat, to identify potential regulators of salinity stress tolerance [211]. The study revealed the presence of 57,921 unigenes from 53.15 million clean reads, of which 544 were DEGs. The analysis further identified several salt-tolerant genes that encoded abiotic stress-related transcription factors (TFs), heat shock proteins, phosphatases, ATP-binding cassette (ABC) transporters, and PKs [211]. Liu et al. (2020) performed an investigation of RNA-Seq on *Ipomoea pes-caprae* to examine its regulatory networks for salinity stress tolerance [214]. It was shown that *I. pes-caprae* contained unique genes associated with the tolerance of salt stress. The findings indicated 40,525 genes, out of which 3334 genes in leaves and 2478 genes in roots were differentially expressed under salt stress. The *ABI2*, *HAI1*, and *EBF1* genes were upregulated in the salt-stressed *I. pes-caprae*. Furthermore, it was found that several candidate genes among the DEGs played a role in hormone signal transduction, and in the signaling pathway of abscisic acid (ABA) and the MAPK for salt stress tolerance. In addition, Zhou et al. (2016) conducted a transcriptomic profile to investigate the salt stress tolerance of the wild progenitor of rice, *O. rufipogon* [215]. The study showed that 6867 transcripts were differentially expressed in different tissue, among which 3105 and 2216 were upregulated in roots and leaves, respectively. Different salt tolerant genes were identified to be co-localized on salt stress tolerance linked loci indicating them as potent genes for the tolerance of salt stress in rice. Several salt-tolerant genes such as *ZFP179*, *ZFP182*, *ZFP252*, *SNAC1*, *SNAC2*, *OsNAC5*, *ONAC045*, *OsACA6*, *OsMYB2*, *OsbZIP23*, *OsBIERF3*, *OsHKT1*, *OsLEA3-2*, *RSOsPR10*, *OsTZF1* were upregulated in *O. rufipogan* during the salinity stress condition. In another study, RNA-Seq was performed from the leaves of *H. spontaneum*, wild barley exposed to NaCl stress, which generated 115 million reads. An enhanced expression of DEGs was found to control various biological activities including ROS scavenging, protein refolding, signaling network, flavonoid biosynthesis, ethylene production, and electron transport under salinity stress [208].

In *G. davidsonii*, a salt-tolerant wild relative of cotton, Fan et al. (2015) identified 109 *WRKY* genes from transcriptomic analyses, which were involved in its salinity stress tolerance [133]. Furthermore, soybean CWRs have displayed an enhanced level of soil salt stress tolerance. It has been found that in wild soybean (accession PI483463), a single dominant gene played a significant role in its tolerance to salinity [142]. It was reported that the *GsWRKY20* gene isolated from *G. Soja*, which encodes for the WRKY-type TF, was capable of enhancing the salinity stress tolerance of transformed susceptible alfalfa plants. Relatively reduced permeability of the membrane and lower content of malondialdehyde content were found in the transformed alfalfa. However, the transformed plants had higher free proline and soluble sugar accumulation [216]. Moreover, the overexpression of the *GsJAZ2* gene from *G. soja*, in *A. thaliana* improved its salinity stress tolerance [217].

Transcriptomic profiles using RNA-Seq were undertaken to compare *H. spontaneum*, a salt-tolerant wild barley genotype, and *Hordeum vulgare*, a salt-sensitive cultivated barley genotype subjected to salt stress [218]. In the salt-tolerant wild plant, a total of 6048 DEGs were found with 3025 up- and 3023 downregulated genes in salt stress conditions. The transcripts of salt stress-related genes were profoundly lower in the salt-sensitive cultivar, which had a total of 2610 DEGs with 580 up- and 2030 down-regulated genes. It was found that the genes, which encoded calcium-binding protein elements, protein kinases, and serine/threonine protein kinases such as *HvCML31*, *HvCML58*, *HvCaMBP1*, *HvSnRK1alpha2*, *HvSnRK1alpha3*, *HvSnRK1beta3*, *HvSnRK3*, and *STKs* were significantly upregulated in the tolerant genotype. The gene ontology (GO) enrichment analysis displayed that the DEGs were mainly linked with stress defenses such as biosynthesis of hormones, scavenging ROS, osmotic homeostasis, regulatory proteins, ion transporters, cellular component, and signaling network [218]. Furthermore, using the short reads sequencing technology (Illumina), the wild *Reaumuria trigyna* plant transcriptome analysis was performed in response to salt stress. By comparing the transcriptomes from control and salt-stressed plants, 5032 genes showed significantly different transcript abundance under salt stress. The transcription profiles revealed that these genes were related to ion transport and the ROS scavenging system that were essential for the morphological and physiological characteristics of wild-tolerant species [219].

A transcriptomic analysis was performed between the wild-type salt-tolerant tomato genotype, *S. pimpinellifolium*, and the cultivated tomato *S. lycopersicum* using Affymetrix Tomato Genome Array containing 9200 probe sets. After the treatment with 200 mM NaCl, a gene encoding for salicylic acid-binding protein 2 (SABP2) was accumulated only in *S. pimpinellifolium*, indicating a potential role for salicylic acid signaling in its salinity stress tolerance. In addition, two genes encoding lactoylglutathione lyase were induced only in wild-type plants, along with much higher basal expression of various glutathione S-transferase genes. This indicated an efficient detoxification salinity stress response mechanism for *S. pimpinellifolium* [220]. In another study, a whole transcriptome analysis was performed between a salt-tolerant mutant line, M4-73-30, and its wild type, Zarjou cultivar using the RNA-Seq method during their seedling stage after six hours of exposure to 300 mM NaCl. Transcriptome sequencing produced 20 million reads for each of the sequenced genotypes. It was identified that a total number of 7116 transcripts were differentially expressed. In mutant and wild-type plants, 1586 and 1479 of the obtained transcripts were significantly expressed [221]. It was found that *Rboh*, *ACC synthase*, *HAK*, and *HVP* genes were significantly expressed in salt tolerant mutant genotype. Furthermore, *on Chrysanthemum lavandulifolium*, transcriptomic analysis was performed to analyze its global gene expression under 200 mM salt stress using digital gene expression technology. In total, 2254 DEGs were found with 1418 upregulated and 836 downregulated genes. Several of the candidate genes such as *PP2C*, *MPK9*, *SnRK2*, *PT2*, and *HKT* were upregulated under the salinity stress. The identified genes were mainly associated to proline biosynthesis, signal transduction, ion transport, ROS scavenging, and flavonoid biosynthesis pathways of the salinity stress responses of *C. lavandulifolium* [222].

The stress-responsive genes identified through transcriptomics from various CWRs can be incorporated into molecular breeding techniques. With several advancements in these techniques, genes from the CWRs can be precisely integrated into the genomes of modern crop cultivars for enhancing their yields and salinity stress tolerance.

## 10. Transcriptomics of CWR in Molecular Breeding Technology

**Figure 4:**
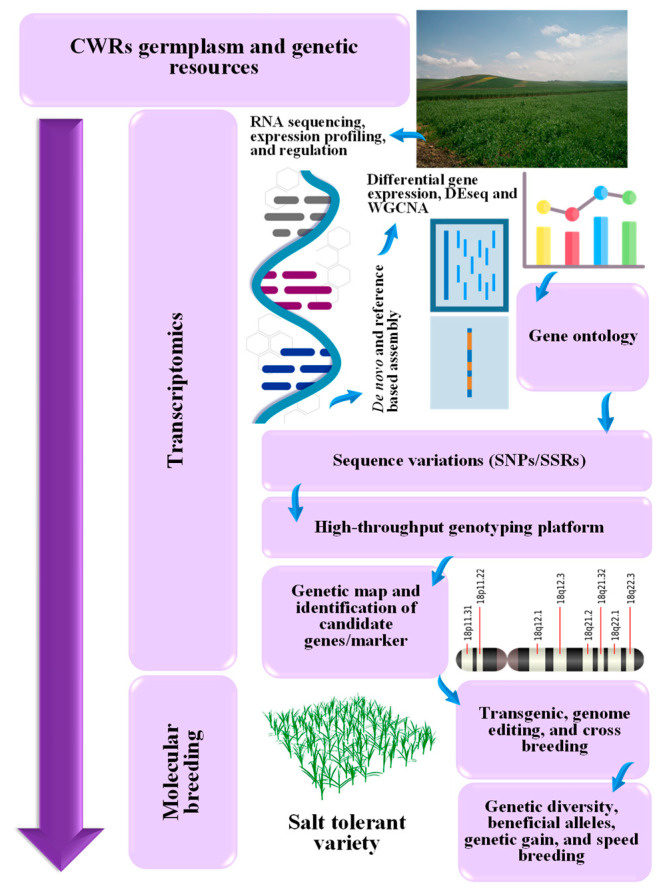
Illustration of the application of transcriptomics in accumulating salt-tolerant alleles in plant genomes for designing tolerant crops. Wild relative germplasm collections in gene banks contain the salt-tolerant beneficial alleles. Transcriptomics through high-throughput sequencing in combination with field phenotyping links the salinity stress tolerance genomic and phenotypic variations. With the identification of beneficial gene trait association, molecular breeding is performed using the wild relatives that will improve the genetic gains of salt stress tolerance breeding program.

The modern molecular approaches utilize the advances in transcriptomics to overcome long-standing obstacles for designing salt stress-tolerant crops using the CWRs. Transcriptomics provides an efficient way of exploring the genetic diversity in the wild relatives of crops to retrieve salt stress tolerant candidate genes or QTL (Figure 4). RNA-Seq or microarrays of wild relatives, followed by de novo assembly can develop reference assemblies that reinforce the application of downstream processes, involving the characterization of stress-tolerant gene’s function [223]. Although it lags behind the cultivated crop transcriptomes, a number of CWRs transcriptomic profiling are analyzed and genome assemblies are being developed [3]. The development of superior assemblies using the third-generation sequencing of long reads has standardized the major crop reference genomes and has built high-quality long read assemblies in wild relatives of crops [224]. Transcriptomics reveal DEGs involved in salt stress tolerance. Furthermore, weighted gene coexpression network analysis (WGCNA) and GO indicate the involvement of salinity stress-responsive genes in various physiological and biochemical processes. In addition, sequence variations analysis, high throughput sequencing, and genetic mapping lead to the accurate identification of salt stress-responsive genes and their location in the CWRs genome. This will reveal the beneficial CWRs genetic diversity to be used for the salinity stress tolerance improvement using modern genomic editing and breeding techniques (Figure 4). Recently, transcription activator-like effectors (TALEs)-based editors were developed successfully to precisely edit the DNA within living cells. In this process, binding is targeted to certain sequences of DNA for amino acid repeats of TALE protein that identify specific bases by a set of biochemical code and activates gene [225]. For molecular breeding, these codes can be used to develop TALEs and TALE protein fusions to bind to any desired DNA sequence for the expression of salt-tolerant genes. TALEs are found to be less toxic and easier to generate as it recognizes DNA nucleotide using a TALE repeat with the corresponding repeat-variable di-residue (RVD) [226]. Artificial TFs have been developed by the fusion of TALE repeat arrays to transcriptional regulatory domains that activated or repressed gene expression [227]. To date, several studies have reported that TALE-based activators and repressors can be used to manipulate the expression of endogenous genes in plants for biotic stress tolerance [226]. However, there are few studies on their uses for the development of salinity stress tolerance in plants using CWRs. Thus, large-scale, systematic studies should prospectively focus on whether adherence to these artificial TFs generated through TALENs from CWRs influences the activities and specificities of cultivated plants’ salt stress tolerance.

The wild-derived genes controlling the salinity stress tolerance can be transferred through a precise genome editing technique, clustered regularly intraspaced short palindromic repeats (CRISPR), into cultivated varieties with less introgression time [228]. This technique is known as de novo domestication which produces new crops using a CWR in a matter of generations utilizing the genome editing tools [228]. De novo domestication of wild *S. lycopersicum* was attained by manipulating its six gene loci that improved the yield, fruit size, and nutritional value of the tomato [229]. Furthermore, allotetraploid rice wild relative has broad genetic diversity that comprises genes involved in several abiotic stress tolerance and higher biomass production in comparison to cultivated rice. However, due to its low grain quality, poor yield, and easy seed shattering, it is not possible to cultivate allotetraploid wild rice as a staple crop [230]. The de novo domestication of allotetraploid wild rice, *Oryza alta*, was recently conducted by targeting six potential agronomically essential genes including abiotic stress, yield, and quality [231]. The results indicated that through the efficient transformation techniques for allotetraploid wild rice, the six agronomically essential traits were successfully domesticated into *O. alta*, and the undesirable characteristics were prevented from occurring. De novo domestication of wild Oryza shows the evidence of transformation of an allotetraploid into a new cereal crop. This can be practiced by other members of crop species for generating abiotic stress resilience. Due to salinity stress tolerance being a multi-genic trait regulated by various TFs, it may be necessary to simultaneously target the TFs of the genes that govern the mechanism of salinity stress tolerance. Thus, de novo domestication can be a promising technique to develop salt stress tolerance in crops, by targeting the transcripts and genes at the same time.

Currently, available commercial genetically modified (GM) crops are dependent on characteristics controlled by a single gene [232]. Due to the multigenic nature of salinity stress, TF-based genetic engineering is a more practical process than the techniques of conventional breeding. It generates an alternative way of enhancing crop salt stress tolerance due to its role in the regulation of stress-responsive genes. Various studies have displayed the role of TFs as transgenes in enhancing plant responses to different stresses within the field and greenhouse conditions using traditional breeding techniques [233]. However, without the use of precise genome editing, beneficial characteristics from CWRs that are introduced into common cultivars using traditional breeding often cause the transfer of unwanted deleterious alleles. A potential technique for overcoming this in regenerated plants is speed breeding [234]. The method of speed breeding involves the manipulation of the photoperiod and temperature in a controlled environment to produce multiple crop generations per year [235]. The use of speed breeding accelerates the timeframe required to fix the genetic background of cultivars, a process that typically requires several years of inbreeding. Speed breeding has been used to efficiently produce several generations in a given year for various crops such as wheat, rice, pea, soybean, and sorghum [236,237,238,239]. Thus, speed breeding can facilitate the rapid growth of several generations, enabling the selection and prevention of undesirable traits, and attaining of stable tolerant genetic varieties.

In addition, the technique of speed breeding benefits the other methods to domesticate the CWRs with desired TFs of salinity stress tolerance without utilizing CRISPR, such as the conversion of germplasm [240]. Germplasm conversion includes its modulation by crossing, selecting various traits in multiple rounds, and inbreeding for adaptation to harsh environments while having desirable agronomic traits [241]. This technique can be used as an alternative to genome editing for transferring essential agronomic traits into wild relatives of crops using hybridization and marker-assisted selection (MAS). The benefit of using germplasm conversion over genome editing is that it does not require specific knowledge of the target sequences, only the TF region that confers the domestication trait is necessary. However, this method is more likely to be laborious and time-consuming in contrast to the techniques of genome editing, as to achieve the final products, several generations are required [242]. Thus, exposing the CWRs to speed breeding conditions will potentially overcome the time-consuming process needed for cycling of multiple generations, which is essential for the efficient conversion of wild relative germplasms into economically viable crops.

## 11. Conclusions

The CWRs’ salinity stress tolerance involves complex reactions at the molecular and cellular levels. These plants harbor stress-responsive genes, which are governed by several TFs such as MYB, bZIP, bHLH, NAC, ERF/AP2, and WRKY. Modern high-throughput sequencing tools have enabled the development of new methods for mapping and quantifying plant transcriptomes. RNA-Seq and microarrays are becoming the preferred method for profiling transcriptome and gene expression in CWRs. These transcriptomics technologies have revealed different genomic regions or specific stress-responsive genes that are involved in the perception and signal transmission of salt stress tolerance in CWRs. In parallel, the sequence information of the CWR genome in combination with precise molecular breeding techniques offers a rapid-track approach to convert cultivated plants into elite salt stress-tolerant crops. These processes can aid in expanding the species diversity range in agricultural production systems, thereby buffering the effects of climatic changes.

## Figures and Tables

**Table 1:** Salt tolerant wild relatives of various crop species.

Wild Species	Plants	Salt Stress Tolerance QTLs/TFs/Genes	Source
*Gossypium tomentosum*	Cotton	*qRL-Chr16-1*	[131]
*Gossypium davidsonii*		*KUP1*, *KUP2*, *KUP11*, *SKOR*, *NCED3*, *PYR/PYL/RCARs*, *SnRK2s*, *AtMYB20*, and *PP2C*	[132]
*Gossypium aridum*		*GarWRKY17* and *GarWRKY104*	[133]
*Solanum hirsutum*	Tomato	*aox-c6.1*, *fla6.1*, *cat-c12.1*, *pox-s7.1*, *pox-s12.1*, *phe9.1*, *phe11.1*, *phe-c2.2*, *phe-c8.1*	[134]
*Solanum parviflorum*		*DREB1A* and *Vp1.1*	[135]
*Arachis diogoi*	Groundnut	*AdDjSKI*	[136]
*Arachis duranensis*		*AdNACs*	[137]
*Arachis glabrata*		*MYB*, *AP2*, *GRAS*, *bHLH*, *C3H*, *WRKY*, *C2H2* and *ARF*	[138]
*Ipomoea imperati*	Potato	*AP2/EREBP*, *bHLH*, *HD-ZIP* and *MYB*	[139]
*Oryza rufipogon*	Rice	*qST1-1*, *qST5-1*, *qST5-2*, *qST9*, *qST10*, *qST11-1*, *qST11-2*, *qST12*, *qST1-1*, *qST1-2*, *qST7*, *qST9*, *qST10*, *qST11-1*, *qST11-2*, *qST12*	[140]
*Tripsacum dactyloides*	Maize	*CH3*, *MYB*, *HB*, *SNF2*, *AUX*, and *SET*	[141]
*Glycine soja*	Soybean	*Ncl2*	[142]
*Medicago ruthenica*	Alfalfa	*NAC*, *C2H2*, and *CAMTA*	[143]

**Table 2:** Overview of the genome assemblies of the wild relatives of several crop species.

Wild Relatives	Plant	Assembly Size (Mb)	Number of Genes	Source
*Glycine soja*	Soybean	1013.2	89,477	[173]
*Hordeum spontaneum*	Barley	4280	725	[174]
*Solanum pennellii*	Tomato	942	32,273	[149]
*Solanum pimpinellifolium*		811	25,970	[150]
*Solanum pennellii*		~1000	-	[175]
*Solanum chilense*		914	25,885	[176]
*Solanum pimpinellifolium*		808.1	35,761	[177]
*Triticum Urartu*	Wheat	3900	34,879	[178]
*Triticum turgidum*		10100	62,813	[179]
*Aegilops tauschii*		4300	39,622	[180]
*Oryza brachyantha*	Rice	261	32,038	[181]
*Oryza meridionalis*		446.4	21,169	[182]
*Oryza rufipogon*		380.5	34,830	[183]
*Oryza granulate*		736.7	40,131	[184]
*Oryza rufipogon*		384.8	22,035	[182]
*Oryza longistaminata*		351	34,389	[185]
*Oryza glaberrima*		316	33,164	[186]
*Oryza officinalis*		584	29,930	[187]
*Oryza rhizomatis*		559	32,083	[187]
*Oryza granulate*		777	40,116	[188]
*Oryza rufipogon*		399.8	36,520	[189]
*Oryza eichingeri*		471	31,030	[187]

## Data Availability

Not applicable.
